# The Effect of Wearing Eyeglasses on the Perception of Attractiveness, Confidence, and Intelligence

**DOI:** 10.7759/cureus.23542

**Published:** 2022-03-27

**Authors:** Saif Aldeen AlRyalat, Mohammed Jumaah, Sari W Al Hajaj, Faisal Al-Noaaimi, Yazan Alawneh, Asad Al-Rawashdeh

**Affiliations:** 1 Ophthalmology, The University of Jordan, Amman, JOR; 2 Internal Medicine, The University of Jordan, Amman, JOR; 3 Surgery, California Institute of Behavioral Neurosciences & Psychology, Fairfield, USA; 4 Nephrology, Texas Tech University Health Sciences Center, Lubbock, USA

**Keywords:** eyeglasses, perception, attractiveness, confidence, intelligence

## Abstract

Introduction

Several studies investigated the effect of wearing eyeglasses on self-esteem measures; however, most of these studies were conducted on western populations. We aim to assess the perception of attractiveness, confidence, and intelligence of young people of college-going age with and without glasses among university students.

Methods

This was a cross-sectional study conducted in five main Jordanian universities. We designed a survey with photos of four people with and without glasses (a total of eight photos). Participants rated the photos on a scale of 10 regarding attractiveness, confidence, and intelligence.

Results

A total of 517 participants were included in this study. We found significantly higher ratings for all domains of pictures without glasses compared to the same pictures with glasses. Moreover, participants not wearing glasses provided significantly higher attractiveness scores for most pictures not wearing glasses.

Conclusion

In our study on Jordanian college students of Arabian ethnicity, we found that eyeglasses may have a negative impact on a person’s image in regard to attractiveness, confidence, and intelligence.

## Introduction

The global prevalence of refractive error is increasing significantly throughout the previous decades, for example, the prevalence of myopia almost tripled in the last 30 years [1]. The importance of correcting refractive error with eyeglasses is well known, and it was proven to enhance the vision-related quality of life [2]. Sight correction with eyeglasses carries associated social and personality effects on the wearer. Studying these effects may provide an insight into how to increase compliance with these corrective devices [3]. During the early 1990s, Terry provided in-depth reviews on social traits affected by eyeglasses, which was followed by several projects that pointed to the importance of the impact of eyeglasses on attractiveness, the degree of confidence, and the degree of intelligence a person is viewed with and without eyeglasses [4-6]. The social and personality effects of wearing eyeglasses are dependent on age, gender, educational level, and other demographic factors that should be considered in assessing eyeglasses’ social and personality impact [6]. Previous studies assessed the impact of wearing eyeglasses on different self-esteem measures in people from western societies [7-10]. However, no previous study assessed the social impacts of wearing eyeglasses in Arabic populations, especially college students. We aim to assess the perception of attractiveness, confidence, and intelligence of young Jordanian people of college-going age with and without glasses among university students wearing and not wearing glasses.

## Materials and methods

This was a cross-sectional study conducted in five main Jordanian universities distributed across Jordan, including The University of Jordan, Jordan University of Science and Technology, Hashemite University, Mutah University, and Balqa Applied University during May, June, and July 2020.

Participants

We distributed the survey to college students through their social media accounts at each university. We included students from all bachelor’s degree faculties at the included universities. We excluded participants who were not able to complete the survey. We used an online service (Google Forms, Google, Mountain View, CA) to collect responses, where we first explained the process and obtained approval from the participants to share the data in the current project.

Assessment

We designed an English language questionnaire that first obtained demographic (i.e., age and gender) and educational factors (i.e., level of education), in addition to asking participants about wearing glasses. After that, photos of four people with and without glasses (a total of eight photos) appeared. An image of one person appeared with or without glasses, then the participant was asked to rate the person in the photo on a scale from 1 (least) to 10 (most) (i.e., visual analog scale) regarding attractiveness (beauty), confidence, and intelligence (smartness). Then the second image appeared and the participant rated it on three domains and so on. The images were shuffled, where an image of one person was followed by an image of a different person so that the image of a person with or without glasses will appear again after five to six images. We obtained photos used in the experiment from Pexels [11], a freely available database for photos, where we used two photos of males and two photos of females with Arabian features and of college-going age. An expert optician decided to choose the most suitable eyeglass based on the shape of the face, the color of the skin, and gender [12]. After choosing the best frame type, we used image simulation programs available at online eyeglass shops to fit the eyeglass with the face [13], thus, creating an image of the same face with an eyeglass. The rationale behind adding glasses via simulation rather than choosing images with and without glasses is to standardize face and ambient-related factors, including the face itself (if images for different people were to be used), the degree of smile, ambient illumination, and so on.

Statistical analysis

We used SPSS version 21.0 (IBM Corp., Armonk, NY) in our analysis. We used the mean (± standard deviation) to describe continuous variables (e.g., age). We used the count (frequency) to describe other nominal variables (e.g., gender). We performed a paired sample t-test to analyze the difference in ratings for the same pictures with and without glasses.

Moreover, we used an independent sample t-test to analyze the difference in ratings for pictures (with and without glasses) with gender, wearing glasses, and history of refractive surgery. We also calculated the reliability of the questionnaire using Cronbach’s alpha index. Parametric analysis assumptions were met. To account for multiple testing, we used Bonferroni correction. We adopted a p-value of 0.05 as a significant threshold.

## Results

A total of 517 participants were included in this study, with a mean age of 22.02 (±1.85) years. There were 182 (35.2%) men and 335 (64.8%) women. Of participants, 346 (66.9%) did not wear glasses. The majority of the included sample (88.2%) did not undergo refractive surgery. The questionnaire had high reliability, as found by Cronbach’s alpha reliability index (Cronbach’s alpha = 0.930).

Table 1 shows the rating scores for the images with glasses and those without glasses. We found a significant difference between the two groups' scores in all images in regard to attractiveness and confidence and in most images in the intelligence aspect (i.e., not significant for one male). Higher ratings were documented for pictures without glasses compared to those same images with glasses. The difference in ratings was higher in magnitude for female images compared to male images, as shown in Table 1.

**Table 1:** Scores for pictures with and without glasses. The difference here represents the mean difference in rated scores with and without glasses for the same person, in the three domains.

		Without glasses: mean (SD)	With glasses: mean (SD)	Difference (95% CI)	t	df	P-value
First male	Attractiveness	8.08 (1.24)	6.68 (1.75)	1.40 (1.23-1.56)	16.67	516	<0.001
	Confidence	8.07 (1.16)	6.33 (1.78)	1.75 (1.57-1.92)	19.73	516	<0.001
	Intelligence	8.14 (1.34)	8.02 (1.63)	0.11 (-0.02-0.25)	1.63	516	0.104
Second male	Attractiveness	8.27 (1.33)	6.71 (1.81)	1.56 (1.39-1.74)	17.40	516	<0.001
	Confidence	8.18 (1.30)	6.26 (1.79)	1.92 (1.74-2.11)	20.17	516	<0.001
	Intelligence	8.28 (1.34)	8.10 (1.64)	0.17 (0.03-0.31)	2.44	516	0.015
First female	Attractiveness	8.97 (1.21)	6.74 (1.80)	2.23 (2.05-2.40)	24.88	516	<0.001
	Confidence	8.74 (1.35)	6.42 (1.94)	2.33 (2.14-2.52)	24.04	516	<0.001
	Intelligence	8.76 (1.36)	8.06 (1.54)	0.70 (0.56-0.84)	9.76	516	<0.001
Second female	Attractiveness	8.52 (1.31)	6.56 (1.87)	1.97 (1.79-2.14)	22.51	516	<0.001
	Confidence	8.44 (1.88)	6.16 (1.88)	2.28 (2.09-2.47)	23.75	516	<0.001
	Intelligence	8.52 (1.29)	8.05 (1.71)	0.47 (0.34-0.62)	6.34	516	<0.001

Regarding factors affecting the difference in scores rated between images with glasses and images without glasses, we found a significant effect of the rater’s status of wearing glasses on attractiveness scores regarding different aspects (Table 2).

**Table 2:** Difference in scores between pictures for people with glasses minus scores for people without glasses, and the difference in rating for rater’s status of wearing glasses. * Difference in attractiveness score between raters without and raters with glasses for the same picture. ** Difference in attractiveness score between pictures without glasses and pictures with glasses in regard to the magnitude of difference for raters (i.e., difference 1).

		Rater wearing glasses: mean (SD)		Difference 1* (95% CI)	P-value	Difference 2** (95% CI)	t (df)	P-value
		No	Yes					
First male attractiveness	Pictures without glasses	8.22 (1.13)	7.80 (1.40)	0.43 (0.19 to −0.67)	0.001	0.64 (0.30-0.97)	3.77 (515)	<0.001
	Pictures with glasses	6.62 (1.77)	6.82 (1.71)	−0.21 (−0.53 to 0.11)	0.203			
Second male attractiveness	Pictures without glasses	8.37 (1.23)	8.09 (1.51)	0.28 (0.02-0.54)	0.037	0.51 (0.16-0.86)	2.84 (515)	0.005
	Pictures with glasses	6.19 (1.84)	6.38 (1.68)	−0.23 (−0.56 to 0.10)	0.175			
First female attractiveness	Pictures without glasses	9.07 (1.10)	8.75 (1.40)	0.12 (0.08-0.56)	0.010	0.77 (0.40-1.13)	4.09 (515)	<0.001
	Pictures with glasses	6.59 (1.75)	7.04 (1.87)	−0.45 (−0.78 to −0.12)	0.007			
Second female attractiveness	Pictures without glasses	8.68 (1.12)	8.22 (1.59)	0.46 (0.19-0.73)	0.001	0.64 (0.28-1.00)	3.48 (515)	0.001
	Pictures with glasses	6.50 (1.84)	6.68 (1.93)	−0.18 (−0.52 to 0.17)	0.308			

Participants not wearing glasses provided significantly higher attractiveness scores for most images not wearing glasses (p-value ranged from 0.037 to 0.001), with higher mean difference scores ranging from 0.12 to 0.46, as shown in Table 2. On the other hand, raters who wore glasses provided non-statistically significant higher scores for images with glasses, with p-values only reaching up to 0.007 for the first female image, with a mean difference of −0.45 (−0.78 to −0.12), a value that lost its significance after correction. Upon comparing the magnitude of difference for without and with glasses images (i.e., difference 2 in Table 2), we found that this magnitude was significantly higher for most images for raters not wearing glasses (p-value ranged from 0.005 to <0.001), with a higher mean difference score ranging from 0.51 to 0.77, as shown in Table 2. No significant difference in scores for age or gender was noted.

## Discussion

Previous studies have pointed to the high impact of eyeglasses on facial image perception and on changing a person's image, where they showed that wearing glasses affect the ability to recognize a person compared to the same person with glasses [7,9]. Such impact of eyeglasses has been poorly assessed in developing countries. Our study included Jordanian college students and assessed their perception regarding the impact of eyeglasses. Participants rated images without glasses as being more attractive, intelligent, and more confident compared to similar images with eyeglasses. Moreover, participants wearing glasses provided higher scores for images with glasses, whereas participants not wearing glasses did not provide higher scores for images without glasses.

Most of the previously conducted studies were done on western samples using images from their societies and cultural norms, and their findings were variable. Previous studies assessing the impact of eyeglasses on self-image showed the negative impact of eyeglasses on attractiveness [5,10], and this was consistent with our study. Graham and Ritchie in their recent experiment on British participants did not find a significant difference in social traits, including attractiveness, between faces with and without eyeglasses [7]. The shape of the face and the eyeglasses were among the factors that influenced the judgment of attractiveness [10,14].

The effect of wearing eyeglasses on intelligence perception varied among different areas and ethnicities. While our study showed a negative impact of wearing eyeglasses on intelligence rating, studies on western populations found a positive impact on intelligence perception for images and people wearing eyeglasses [4,10,15], where this variability is probably due to different cultural associations with wearing eyeglasses [15]. In a study conducted in France, people with eyeglasses were considered of higher socio-professional status than without glasses [16]. Another study found a positive impact of eyeglasses on one’s image of competency and warmth [17]. The positive impact of eyeglasses on intelligence perception is also found in older studies on the western population [5]. In a study assessing intelligence level among a large cohort with different refractive errors, the authors found a higher level of intelligence and education among myope compared to no refractive error. [18] Compared to western studies where eyeglasses have a positive impact on the intelligence image of a person, our study that included participants of Arabian ethnicities rated images with glasses with lower intelligence scores compared to images without glasses, which might represent a sort of social stigma related to wearing glasses among Jordanians.

The present study has different aspects of strength, including the use of an objective method of assessing eyeglasses' effect on images (i.e., visual analog scale on simulated images), and having a representative sample of Jordanian college students from universities distributed across Jordan. We also believe our study has several limitations that should be considered in future projects: we did not assess the effect of eyeglasses rim, facial features, and other factors that may influence the perception of attractiveness. Image simulation may also introduce certain artifacts that may not be present in real-life situations, such as observing faces from the front only in simulated images. Moreover, using the same image twice (i.e., one with and then without glasses), will bias the judgment of the rater toward giving a similar score. However, shuffling the images will yield a lag period of around three to four minutes before the next similar image appears, thus, decreasing the bias discussed.

## Conclusions

The effect of eyeglasses has a different impact on the social traits of wearers, depending on social and ethnic variables. In our study on Jordanian college students of Arabian ethnicity, we found that eyeglasses may have a negative impact on a person’s image in regard to attractiveness, confidence, and intelligence. Moreover, participants wearing glasses provided higher scores for images with glasses, whereas participants not wearing glasses did not provide higher scores for images without glasses.
